# Collection and curation of prokaryotic genome assemblies from type strains at NCBI

**DOI:** 10.1099/ijsem.0.005707

**Published:** 2023-01-19

**Authors:** Sivakumar Kannan, Shobha Sharma, Stacy Ciufo, Karen Clark, Seán Turner, Paul A. Kitts, Conrad L. Schoch, Michael DiCuccio, Avi Kimchi

**Affiliations:** ^1^​ National Center for Biotechnology Information, National Library of Medicine, National Institutes of Health, 9600 Rockville Pike, Bethesda, MD, 20892, USA

**Keywords:** genome, taxonomy, type material, type strain, ANI, GenBank

## Abstract

The public sequence databases are entrusted with the dual responsibility of providing an accessible archive to all submitters and supporting data reliability and its re-use to all users. Genomes from type materials can act as an unambiguous reference for a taxonomic name and play an important role in comparative genomics, especially for taxon verification or reclassification. The National Center for Biotechnology Information (NCBI) collects and curates information on prokaryotic type strains and genomes from type strains. The average nucleotide identity (ANI)-based quality control processes introduced at NCBI to verify the genomes from type strains and improve related sequence records are detailed here. Using the curated genomes from type strains as reference, the taxonomy of over 1.1 million GenBank genomes were verified and the taxonomy of over 7000 new submissions before acceptance to GenBank and over 1800 existing genomes in GenBank were reclassified.

## Data summary

Detailed descriptions of each file and the file contents are provided in the corresponding README file in the same directory. The following files are part of the public NCBI Taxonomy dump files (https://ftp.ncbi.nlm.nih.gov/pub/taxonomy/new_taxdump):

1. excludedfromtype.dmp – a manually curated list of strains that were listed incorrectly as type strains in the literature or other public resources.
2. typematerial.dmp – list of type materials along with the corresponding NCBI Taxonomy numeric-identifier (TaxId) and whether the type material is from a heterotypic synonym.

The following files are part of the public NCBI Genomes FTP files (https://ftp.ncbi.nlm.nih.gov/genomes/ASSEMBLY_REPORTS):

1. ANI_report_prokaryotes.txt – This file contains ANI data for all latest prokaryotic genome assemblies in GenBank.
2. prokaryote_type_strain_report.txt – This file provides information about every prokaryote in NCBI Taxonomy that has a type strain and/or a genome assembly (of type and/or non-type strains).
3. prokaryote_without_type_assembly.txt – This file provides information about prokaryotes in NCBI Taxonomy that have type strains but do not have assemblies available from type strains.
4. prokaryote_ANI_type_not_matching.txt – This file provides information about assemblies from type strains that appear to be significantly different from the other assemblies available for the same organism.
5. prokaryote_ANI_species_specific_threshold.txt – This file provides species-specific ANI thresholds for species that do not use the default ANI threshold of 96 % to define species boundaries.

## Introduction

### Type materials

The public sequence databases are confronted with the dual charge of providing an accessible archive to all submitters and supporting data reliability and its re-use to all users. This makes improving the trustworthiness and provenance of public records a long-standing challenge. The databases in the International Nucleotide Sequence Database Collaboration [INSDC, whose members are GenBank, the European Nucleotide Archive (ENA) and the DNA Databank of Japan (DDBJ)] [1] as well as additional resources at the National Centre for Biotechnology Information (NCBI) such as RefSeq [2] use a central classification and nomenclature resource – the NCBI Taxonomy [3]. This resource is structured around formalized taxonomic names governed under several codes of nomenclature that extensively lay out how names and species descriptions are published. The codes also define how to document a ‘type’, an element (usually a specimen or culture) to which the name of a species is permanently attached.

The introduction of type material annotations within NCBI Taxonomy in 2014 marked an important development in how taxonomic assignments can be validated in NCBI databases [4]. This unlocked the ability to implicitly connect species names to physical vouchers and public sequence records. Since a sequence obtained from type material can act as an unambiguous reference for a taxonomic name and any associated species concepts, it becomes possible to compare and adjust names of closely related records, according to their similarity [5].

The term ‘type material’ as official vocabulary by the INSDC (www.insdc.org/controlled-vocabulary-typematerial-qualifer) includes several variations on ‘type’ as defined in the relevant codes, with ‘type material’ collectively referring to all. The International Code of Nomenclature of Prokaryotes (ICNP), governing most bacterial and archaeal names, requires that for each species a designated strain be ‘maintained in pure culture’ that ‘should agree closely to its characters with those in the original description’ [6]. This type strain is required to be placed in at least two publicly accessible culture collections in different countries. These strains are frequently swapped with additional repositories, and all subcultures are subsequently referred to as co-identical type strains. Because of all these actions there is a potential for errors and contaminants to be introduced.

The NCBI Taxonomy group and their colleagues maintain and update published information related to type strains and other type material according to the rules of relevant codes of nomenclature [7]. This results in a list of co-identical type strains attached to validly published names along with their relevant publications. For example, *Kitasatospora aureofaciens* has at least 40 known co-identical type strains listed in NCBI Taxonomy (https://www.ncbi.nlm.nih.gov/Taxonomy/Browser/wwwtax.cgi?id=1894) and therefore a comprehensive comparison for any sequence derived from these type strains is required in order to discover any potential errors.

At NCBI, genome assemblies obtained from type strains (referred to as ‘type assemblies’) are utilized in computational comparisons, e.g., using average nucleotide identity (ANI) to make changes with reasonable confidence, such as reclassifying or modifying existing taxonomy. Like other assemblies, type assemblies are also prone to errors, such as mislabeling, contamination or sequencing issues, but unlike them, these problems will have harmful effects on many other genomes. As a result, particular care is taken when annotating type assemblies. The processes introduced at NCBI to verify the type assemblies and improve related sequence records are detailed here.

## Methods

### Annotating assemblies from type materials

NCBI Taxonomy maintains a manually curated list of validly and effectively published species names as defined by the ICNP [6] and type strains along with the relevant publications. NCBI curators rely on information in publications and important online resources such as the List of Prokaryotic names with Standing in Nomenclature (LPSN) managed by curators at the Leibniz Institute [8]. NCBI Taxonomy also maintains a manually curated list of strains that were listed incorrectly as type strains in the literature or other public resources. The list can be found in the FTP file, excludedfromtype.dmp, which can be found as part of the public NCBI Taxonomy dump files (https://ftp.ncbi.nlm.nih.gov/pub/taxonomy/new_taxdump), detailed in [3]. The strains in this list and assemblies from these strains were excluded from all our analyses. Type strain names along with the corresponding NCBI Taxonomy numeric-identifier (TaxId; specifically indicated as NCBI:txid <number>) and whether the type strain is from a heterotypic synonym was obtained from the FTP file, typematerial.dmp, which can be found as part of the public NCBI Taxonomy dump files. Additional information about the FTP files is available as part of the general taxdump readme file (https://ftp.ncbi.nlm.nih.gov/pub/taxonomy/new_taxdump/taxdump_readme.txt).

Using publicly available search options, a list of GenBank sequence records along with their available metadata, such as the species and the strain name, can be obtained. Common guidelines for source modifiers are not consistently followed by submitters (see: https://www.ncbi.nlm.nih.gov/WebSub/html/help/genbank-source-table.html#modifiers) so strain information can be found either as ‘strain’ or under other modifiers such as ‘isolate’, ‘culture collection’ or ‘specimen voucher’ on a given record. Starting with the list of NCBI TaxIds and type strain information, the corresponding GenBank sequences were identified using an extended Entrez search. Regular expression matches were used to account for minor differences in the type strain names among the Taxonomy and GenBank data (for example, a missing space between the culture collection name and the identifier; ATCC 123 vs ATCC123). Here is a sample Entrez query against the NCBI Nucleotide database (https://www.ncbi.nlm.nih.gov/nuccore) to get all GenBank sequences from the TaxId of *Bacillus pseudomycoides* (NCBI:txid64104) and strain DSM 12442: *”txid64104”[orgn] AND “DSM 12442”[strain]*. NCBI GenBank sequences were then mapped to their corresponding NCBI Assembly.

### Assessing average nucleotide identity (ANI) and species-specific thresholds

On the basis of our previous analyses, an ANI threshold of 96 % was used as a default value to define species boundaries [5]. However, there were cases in which a custom species-specific threshold, higher or lower than the default 96%, was required. A higher threshold was used when the species were closely related, and a lower ANI threshold was used for species with broader genomic diversity. Most custom ANI thresholds were automatically determined and then reviewed and approved by the taxonomy curators at NCBI (see below) based on information from publications. In a few exceptional cases, custom ANI thresholds were chosen based on recommendations from external experts familiar with these species (Fig. S1, available in the online version of this article).

Custom species-specific ANI thresholds were determined as follows:

1. For a given species with at least four assemblies and with at least one from the type strain, get the ANI of all assemblies that match the type assembly or assemblies.
2. Find the minimum ANI among all the matches from the same species (*same-species_min_ANI*).
3. Find the maximum ANI among all the matches from different species (*cross-species_max_ANI*).
4. If *same-species_min_ANI* is greater than *cross-species_max_ANI* and the difference between them is at least 1 % then recommend a value that is 20 % of the difference above *cross-species_max_*ANI (*cross-species_max*_ANI + ((*same-species_min*_ANI - *cross-species_max_ANI*) * 0.20))

### Assemblies not used as types

NCBI evaluates all assemblies, including type assemblies, for potential anomalies: contamination, misassembly, taxonomic misidentification, etc. (for a full list, see https://www.ncbi.nlm.nih.gov/assembly/help/anomnotrefseq/). Assemblies that fail one or more tests are considered out-of-scope for inclusion in RefSeq [2]. Assemblies from type material that fail a subset of the tests were excluded and not used as types (see supplementary information for the list of tests). The following Entrez query against the NCBI Assembly database (https://www.ncbi.nlm.nih.gov/assembly) lists all assemblies from type material that are not used as type and the reason(s) why they are not used as type: *”not used as type”[Excluded from RefSeq]*.

Assemblies from type material that failed some of the tests were still included only if there were no other type assemblies available for the corresponding species (see supplementary material for the list of the tests). However, these type assemblies were not used for making changes such as reclassifying or modifying existing taxonomy of other assemblies. The following query can be used against the NCBI Assembly database (https://www.ncbi.nlm.nih.gov/assembly) to get all type assemblies which belong to this category:

*"from type"[Properties] AND ("derived from metagenome"[Excluded from RefSeq] OR "derived from environmental source"[Excluded from RefSeq] OR "fragmented assembly"[Excluded from RefSeq]).*

### Identifying potentially problematic type assemblies

NCBI uses the ANI method to identify misassigned or contaminated assemblies [5]. In brief, two assemblies are considered matching if their respective coverage is above 80 % and their ANI value is above 96 % (for most species). An assembly that matches a type assembly from a different species is usually considered potentially misidentified. However, for non-type assemblies, if the interspecies matches are from closely related species from the same genus, the submitted species name of the assembly is accepted and not considered misidentified. For type assemblies, stricter criteria were used to ensure that the type assemblies were not misidentified.

A type assembly is considered potentially problematic and flagged for manual review by a curator if any one of the following conditions is true:

1. It doesn’t match other type assemblies from its own species (*missing-type-matches*)
2. It matches a type assembly from the same genus at very high ANI (>98 %) and at least 80 % query and subject coverage (*intra-genus-mismatch*)
3. It matches a type assembly from a different genus above the ANI threshold (usually 96 %) and with at least 75 % query and subject coverage (*inter-genus-mismatch*)
4. At least 50 % of the non-type assemblies from the same species with four or more non-type assemblies do not match the type assembly above the ANI threshold and with 75 % query and subject coverage (*missing-non-type-matches*)

A type assembly is considered to be potentially contaminated with another assembly (or assemblies) if at least either 200 000 bp or 5 % of the query assembly matches a type assembly from a different species with at least 95 % ANI. Assemblies that satisfy these conditions are considered potentially contaminated and marked for manual review.

Following the manual review, a potentially problematic type assembly might be rescued and marked as not problematic. If the type assembly was found to be misassigned or contaminated, it is flagged not to be used as type. This will not only remove a problematic type assembly from the ANI process and the public view, it may also rescue other type assemblies that were considered potentially problematic because of this one. In the NCBI FTP file, ANI_report_prokaryotes.txt (https://ftp.ncbi.nlm.nih.gov/genomes/ASSEMBLY_REPORTS/ANI_report_prokaryotes.txt), the column, ‘assembly-type-category’ indicates if the type assembly is considered potentially problematic. NCBI does not initiate changing an assembly taxonomy using potentially problematic type assemblies as evidence.

## Results and discussion

### Assemblies from type strains

There are more than 20 600 validly published species names listed in NCBI Taxonomy (https://ncbi.nlm.nih.gov/taxonomy) after excluding names below species rank, unclassified, uncultured and those with informal names, as well as effectively published names and *Candidatus* names: *(Prokaryotes[SubTree] AND species[Rank] AND specified[prop] NOT (uncultured[prop] OR ”effective current name“[Filter] OR ”candidatus current name“[Filter])*. When adding the Entrez search term to the above search: *AND ”has type material“[prop]* yields 19 478 that are validly published with annotated type material. In addition, at least 70 % of the taxa with annotated type strains have at least one assembly from type material.

There are at least 18 281 assemblies from type strains from 14 017 taxa with validly published species names (Table 1). In terms of type category, 18 811 of the assemblies are from type strains while 350 of the assemblies are from type strains of species classified as heterotypic synonyms (Table 2). A full list of type assemblies can be obtained with the following Entrez query against the NCBI Assembly database (https://www.ncbi.nlm.nih.gov/assembly): *bacteria[organism] AND (”assembly from type material“[filter] OR ”assembly from synonym type material“[filter] OR ”assembly designated as neotype“[filter] OR ”assembly from pathotype material“[filter])*. Additionally, the type assemblies per taxon count is available as an FTP file (https://ftp.ncbi.nlm.nih.gov/genomes/ASSEMBLY_REPORTS/ prokaryote_type_strain_report.txt).

**Table 1.** Type strains and assemblies from type strains grouped by their publication status

	Validly published	Effectively published*
Number of taxa with type strains	19 478	1592
Number of type and/or co-identical strains†	91 526	4897
Number of taxa with type assembly	14 017	903
Number of assemblies from type and/or co-identical strains	18 281	950

* A taxon that has been described in a journal other than the *International Journal of Systematic and Evolutionary Microbiology* (*IJSEM*) is considered to have an effectively published, but not validly published name.

† Subcultures of the original type strains.

**Table 2.** Taxa and assembly counts from different type categories

	Taxa		Assemblies	
	species	subspecies	species	subspecies
Type strain	14 656	300	18 310	501
Synonym type strain*	231	4	338	12
Pathotype strain†	13	1	54	2
Neotype type strain‡	8	0	18	0

* Type strain from a heterotypic synonym.

† Type strain from a pathovar.

‡ Replacement strain for a type strain that has been lost.

There are at least 2500 species with at least one assembly but none from a type strain. Most of the species without type assemblies are *Candidatus* species. The top ten species with highest number of assemblies but without any assembly from type strains, are listed in Table 3. Additionally, an up-to-date full list is also published as an FTP file (https://ftp.ncbi.nlm.nih.gov/genomes/ASSEMBLY_REPORTS/prokaryote_without_type_assembly.txt). The type strains from the species at the top of the list are high priority candidates for sequencing. The species for which type strains are not available because the strains cannot be cultured, for example, are candidates for manually designated reference type strains.

**Table 3.** Top ten species with highest number of assemblies but without any assembly from type strains

Species	Number of assemblies
* Francisella tularensis *	570
* Eubacterium rectale *	190
* Streptococcus iniae *	95
* Vibrio cyclitrophicus *	91
* Weissella confusa *	88
* Coxiella burnetii *	83
* Providencia stuartii *	69
* Xanthomonas fragariae *	65
* Fusicatenibacter saccharivorans *	61
* Vibrio breoganii *	59

### Assemblies from “type strains” submitted for effectively published taxa and *Candidatus* species

A taxon that has been described in a journal other than the *International Journal of Systematic and Evolutionary Microbiology* (*IJSEM*) is considered to have an effectively published, but not validly published name and has no standing under the ICNP (Rules 25, 27 and 30 [6]). To be considered for ‘validly published’ status, and subsequently be treated as a ‘formal’ name in the NCBI Taxonomy, the effectively published names must be submitted to and included in an *IJSEM* Validation List. However, in a survey in 2018 it was found that 150 such effectively published names per year on average were never submitted for inclusion on the validation lists [9]. Often, it is possible that the only available data for some sparsely sampled lineages are those under effectively published names. In these cases, it is important that the taxa with effectively published names are identified and submitted for validation.

NCBI Taxonomy also maintains the list of taxa with effectively published names and their ‘type strains’. Since effectively published names have no standing in nomenclature, NCBI does not use the ‘type assemblies’ from these taxa to validate taxonomic assignments through ANI. Currently, there are 1500 such taxa and there are at least 900 assemblies from these strains. Of these, there are at least 73 species from 71 genera for which no assembly from any type strain is available (Table 4). Another set of names requires similar attention. *Candidatus* names are submitted for putative taxa of as yet uncultivated prokaryotes under specific conditions [10]. In several cases ‘type strains’ were documented, usually resulting in isolate numbers annotated on sequence records. However, these names have no standing under the current ICNP as they are only addressed in an appendix and these strains are indicated as reference strains only. These assemblies were excluded in this study and no actions or corrections through ANI or otherwise are currently done by comparing these assemblies. However, if a broadly accepted standard arises this will be reassessed in future.

**Table 4.** List of effectively published names* including genera without any validly published species

*Acidibacillus ferrooxidans*
*Acidibacillus sulfuroxidans*
* Acidithrix ferrooxidans *
*Anaeromassilibacillus senegalensis*
* Anatilimnocola aggregata *
*Angelakisella massiliensis*
*Bacilliculturomica massiliensis*
*Bariatricus massiliensis*
*Beduini massiliensis*
* Bittarella massiliensis *
*Colibacter massiliensis*
*Culturomica massiliensis*
*Dakarella massiliensis*
*Dehalobacterium formicoaceticum*
*Desnuesiella massiliensis*
*Duodenibacillus massiliensis*
* Edaphobacillus lindanitolerans *
*Emergencia timonensis*
*Estrella lausannensis*
* Euryhalocaulis caribicus *
*Evtepia gabavorous*
*Ferrovum myxofaciens*
* Fuerstia marisgermanicae *
* Gemmatirosa kalamazoonensis *
*Ghiorsea bivora*
*Hankyongella ginsenosidimutans*
*Humisphaera borealis*
*Intestinibacillus massiliensis*
* Lacunisphaera limnophila *
*Lascolabacillus massiliensis*
*Levyella massiliensis*
*Mailhella massiliensis*
*Marasmitruncus massiliensis*
*Massilibacillus massiliensis*
*Massilibacterium senegalense*
* Massiliimalia massiliensis *
* Massiliimalia timonensis *
*Massilioclostridium coli*
*Mediterranea massiliensis*
*Merdibacter massiliensis*
*Metallococcus carri*
*Metaprevotella massiliensis*
*Methylacidimicrobium cyclopophantes*
*Millionella massiliensis*
*Mobilibacterium timonense*
*Murdochiella massiliensis*
*Natronogracilivirgula saccharolytica*
*Ndongobacter massiliensis*
*Necropsobacter massiliensis*
*Neglecta timonensis*
*Neofamilia massiliensis*
*Niameybacter massiliensis*
*Nigerium massiliense*
*Nissabacter archeti*
* Numidum massiliense *
* Oceanicoccus sagamiensis *
* Ochrovirga pacifica *
* Olegusella massiliensis *
*Paramesorhizobium deserti*
*Parasaccharibacter apium*
*Phocea massiliensis*
*Prevotellamassilia timonensis*
*Provencibacterium massiliense*
*Pygmaiobacter massiliensis*
*Rappaport israeli*
* Rubeoparvulum massiliense *
*Sanguibacteroides justesenii*
*Senegalia massiliensis*
*Superficieibacter electus*
* Tetzosporium hominis *
* Thermanaerothrix daxensis *
*Traorella massiliensis*
*Vaginella massiliensis*

* A taxon that has been described in a journal other than the *International Journal of Systematic and Evolutionary Microbiology* (*IJSEM*) is considered to have an effectively published, but not validly published name.

### Assemblies not used as types

Table 5 shows the reason and the count of type assemblies that failed one or more requirements for an assembly to be considered for inclusion in RefSeq. The three most common problems were, ‘contaminated’ i.e., an unintended mixture of two separate species; followed by ‘unverified source organism’ i.e., the taxonomic assignment of the assembly is misidentified; and ‘fragmented assembly’ i.e., poor sequence data. The following Entrez query against the NCBI Assembly database (https://www.ncbi.nlm.nih.gov/assembly) lists all assemblies from type material that are not used as type and the reason(s) why they are not used as type: *”not used as type”[Excluded from RefSeq]*.

**Table 5.** Reason and count of assemblies that have been excluded as type assemblies, after curation. See supplementary information for an explanation of the reasons

Reason	Count
Contaminated	216
Unverified source organism	204
Fragmented assembly	92
Genome length too large	32
Genus undefined	28
Misassembled	28
Genome length too small	24
Low quality sequence	22
Derived from metagenome	7
Partial	6
Missing strain identifier	1
Derived from single cell	1
Derived from environmental source	1

### Potentially problematic type assemblies

A total of 1033 assemblies were found to be potentially problematic based on the criteria listed in the methods for identifying potentially problematic type assemblies. Table 6 shows the reason and the count of these type assemblies. This list is regarded with more uncertainty than those in Table 5 with not enough information to decide whether they were truly problematic (the suspected type assembly was the only assembly available for its species, for example). A total of 465 type assemblies had either failed to match the other type assemblies from their own species, if available, or matched type assemblies from other species. For example, the type assembly, GCA_001890655.1 from *Vibrio fluvialis* didn’t match the three type assemblies from its own species and matched all the four type assemblies from *Vibrio vulnificus* (Fig. S2). There were at least 22 pairs of taxa that matched types from a different genus (Table 7). There were 126 taxa with one or more type assemblies for which all type assemblies from these taxa were found to be problematic, essentially leaving these taxa with no type assemblies after curation.

**Table 6.** Potentially problematic type assemblies

Type assembly suspect category	Count
missing-type-matches	47
intra-genus-mismatch	389
inter-genus-mismatch	29
missing-non-type-matches	568

**Table 7.** Inter-genus mismatches among type assemblies

Taxon 1	Assembly accession from Taxon 1	Taxon 2	Assembly accession from Taxon 2
* Anaeroarcus burkinensis *	GCA_000430605.1	* Anaeromusa acidaminophila *	GCA_000374545.1
*Arcobacter peruensis*	GCA_003711085.1	* Poseidonibacter parvus *	GCA_001956695.1
* Bacillus cihuensis *	GCA_000504145.1	* Peribacillus huizhouensis *	GCA_014138605.1
* Bombella apis *	GCA_014878255.1	*Parasaccharibacter apium*	GCA_002917995.1
* Bombella apis *	GCA_018221685.1	*Parasaccharibacter apium*	GCA_002917995.1
* Chimaeribacter arupi *	GCA_002858805.1	*Nissabacter archeti*	GCA_900130115.1
*Chryseobacterium manosquense*	GCA_014623485.1	* Kaistella haifensis *	GCA_000735695.2
* Clostridium methoxybenzovorans *	GCA_000421505.1	* Lacrimispora indolis *	GCA_000526995.1
* Companilactobacillus metriopterae *	GCA_004117915.1	* Lactobacillus terrae *	GCA_002762335.1
* Desulfovibrio desulfuricans *	GCA_900142115.1	* Halodesulfovibrio aestuarii *	GCA_000384815.1
* Entomoplasma ellychniae *	GCA_002930155.1	* Mesoplasma corruscae *	GCA_002930145.1
* Gramella jeungdoensis *	GCA_004378585.1	* Lutibacter litoralis *	GCA_014646675.1
* Halopseudomonas gallaeciensis *	GCA_003444685.1	* Pseudomonas abyssi *	GCA_002307495.1
* Macellibacteroides fermentans *	GCA_013409575.1	* Parabacteroides chartae *	GCA_900168155.1
*Mannheimia massilioguelmaensis*	GCA_000940515.1	* Pasteurella bettyae *	GCA_900454515.1
*Mannheimia massilioguelmaensis*	GCA_000940515.1	* Pasteurella bettyae *	GCA_000262245.1
* Mycobacterium kyogaense *	GCA_003254575.1	* Mycolicibacterium obuense *	GCA_001044245.1
*Mycobacterium novum*	GCA_010726505.1	* Mycolicibacter algericus *	GCA_002086455.1
*Mycobacterium novum*	GCA_010726505.1	* Mycolicibacter sinensis *	GCA_000214155.1
* Mycobacterium senegalense *	GCA_019645875.1	* Mycolicibacterium conceptionense *	GCA_001052995.1
* Mycobacterium senegalense *	GCA_019645875.1	* Mycolicibacterium conceptionense *	GCA_002102065.1
* Mycobacterium senegalense *	GCA_019645875.1	* Mycolicibacterium farcinogenes *	GCA_000723385.1
* Ornithinicoccus soli *	GCA_005222685.1	* Segeticoccus rhizosphaerae *	GCA_009192725.1
* Parascardovia denticolens *	GCA_900445765.1	* Scardovia inopinata *	GCA_001042695.1
* Tenebrionibacter intestinalis *	GCA_016632365.1	* Tenebrionicola larvae *	GCA_019148575.1

### Genomic coherence of assemblies from type and/or co-identical strains

**Fig. 1.**
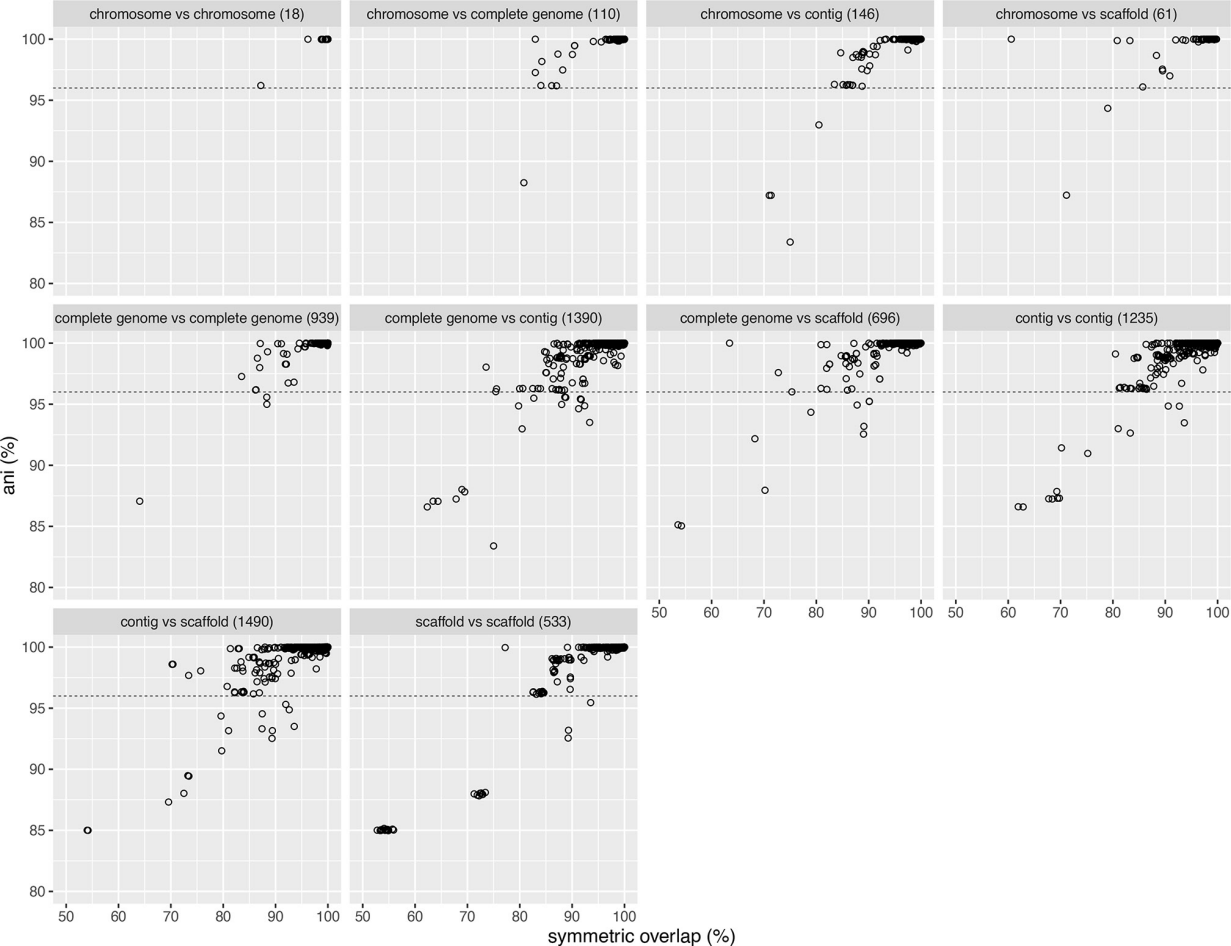
Assemblies from type and/or co-identical strains from the same species were mostly similar with fewer outliers. Genomic coherence or similarity among a pair of type assemblies from the same species was measured using average nucleotide identity (ANI) and symmetric overlap (matched region length over the total length among a pair of assemblies). Assemblies were grouped by their assembly levels (see supplementary information for descriptions of assembly levels) to check if the difference in assembly levels could explain any lack of coherence among type assemblies from the same species. Table 8 lists the extreme outliers.

Assemblies from all co-identical type strains are expected to be identical and ANI can be used for cross verification. At a minimum, these assemblies should reciprocally match each other above the ANI threshold and above 80 % query and subject coverage. Fig. 1 shows the genomic coherence among the type assemblies from the same species. Most of the assemblies from the same species were highly similar (ANI above the threshold) except for the differences in coverage. At least one or more type assemblies from 18 taxa are significantly different from the rest of the type assemblies from their corresponding species (Table 8).

**Table 8.** List of taxa whose assemblies from co-identical strains* differ significantly among themselves (lower ANI values)

Scientific name	Assembly accessions from type strains
* Acidithiobacillus thiooxidans *	GCA_000227215.2; GCA_006718285.1; GCA_009662475.1
* Cereibacter azotoformans *	GCA_003050905.1; GCA_017599285.1
* Chitinophaga alhagiae *	GCA_003177275.1; GCA_003568665.1
* Chryseobacterium potabilaquae *	GCA_902728265.1; GCA_902728305.1
* Granulicatella elegans *	GCA_000162475.2; GCA_020735385.1
* Humibacillus xanthopallidus *	GCA_006716675.1; GCA_006717145.1
*Hymenobacter negativus*	GCA_016056375.1; GCA_017571495.1
* Mycolicibacterium parafortuitum *	GCA_002086815.1; GCA_010725485.1
* Mycoplasma conjunctivae *	GCA_000026765.1; GCA_900660555.1
* Neisseria sicca *	GCA_000174655.1; GCA_014054945.1; GCA_019334765.1
* Piscirickettsia salmonis *	GCA_000297215.2; GCA_000300295.4; GCA_000401515.2
* Priestia megaterium *	GCA_010671665.1; GCA_900113355.1
* Primorskyibacter flagellatus *	GCA_014638275.1; GCA_900176485.1
* Rhodococcus wratislaviensis *	GCA_000583735.1; GCA_900455735.1
* Rhodomicrobium vannielii *	GCA_000166055.1; GCA_016461745.1
* Rubrobacter xylanophilus *	GCA_000014185.1; GCA_019448335.1
* Streptomyces clavuligerus *	GCA_000154925.1; GCA_000163875.1
* Treponema rectale *	GCA_014202035.1; GCA_014984185.1

* Subcultures of the original type strains.

ANI-based curation helps to identify potentially problematic type strains or assemblies from the type strains. However, this can be influenced by various confounding factors, such as accumulated mutations, mislabeling, contamination or sequencing errors. Here are few examples:

1. The strains were mislabeled or switched before or during submission (e.g., *Humibacillus xanthopallidus *, *Mycolicibacterium parafortuitum *)
2. Problematic sequencing or assembly (many frameshifted proteins e.g., GCA_010671665.1 from *Priestia megaterium *)
3. Difference in the submitted assembly with respect to inclusion or exclusion of plasmids (e.g., *Mycoplasma conjunctivae *)
4. Difference in assembly quality (fragmented Whole Genome Shotgun (WGS) genomes vs complete genomes)

### Assemblies from type strains are not always representative of their corresponding species, especially for species with broad diversity

**Fig. 2.**
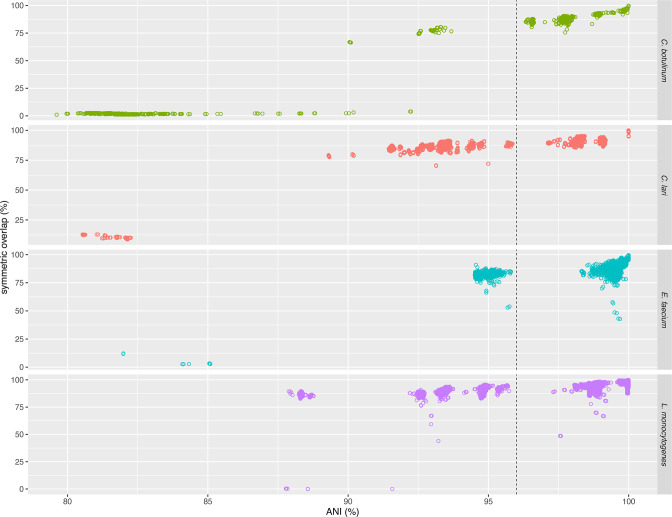
Examples of assemblies from species with high intraspecies genomic diversity. Intraspecies ANI vs symmetric overlap (matched region length over the total length among a pair of assemblies) of assemblies against their corresponding type assemblies from *Clostridium botulinum* (* C. botulinum *), *Campylobacter lari* (* C. lari *), *Enterococcus faecium* (* E. faecium *) and *Listeria monocytogenes* (* L. monocytogenes *). The vertical dashed line indicates the default ANI threshold, 96 %. There are many assemblies in clusters that match their corresponding type assemblies below the expected ANI threshold and/or coverage.

Type assemblies are not necessarily obtained from typical representatives of their associated species as noted in ICNP Rule 15. This is especially evident for species with broader genomic diversity. The type strains just happen to be selected from the first isolates analysed for a novel species. Amongst the 1600 species with at least four non-type assemblies that were examined, more than 50 % of the non-type assemblies in 310 species do not match the type assemblies from their corresponding species above the expected ANI threshold of 96 % (*missing-non-type-matches*; see Methods). A list of species with high intraspecies genomic diversity that were identified in this study can be found here (https://ftp.ncbi.nlm.nih.gov/genomes/ASSEMBLY_REPORTS /prokaryote_ANI_type_not_matching.txt). It is possible that some of these assemblies that do not match their type assemblies were misidentified. In other cases, this reflects a more complex genomic diversity (and undescribed, potential taxa) that was corroborated by literature. For example, there are three biotypes (bt1, bt2 and bt3) for *Vibrio vulnificus* [11], four distinct groups (Group I, II, III and IV) for *Clostridium botulinum* [12] and two genomospecies groups (GS1 and GS2) for *Campylobacter concisus* [13]. In these cases, the type strain does not adequately represent all these subspecific genetically divergent groups that are not formally named. For example, only nine of the 253 non-type assemblies match the type assembly in *Campylobacter concisus *. Fig. 2 shows a few examples of broad intraspecies genomic diversity among assemblies as measured by their ANI and coverage of all non-type assemblies against their corresponding type assemblies.

**Fig. 3.**
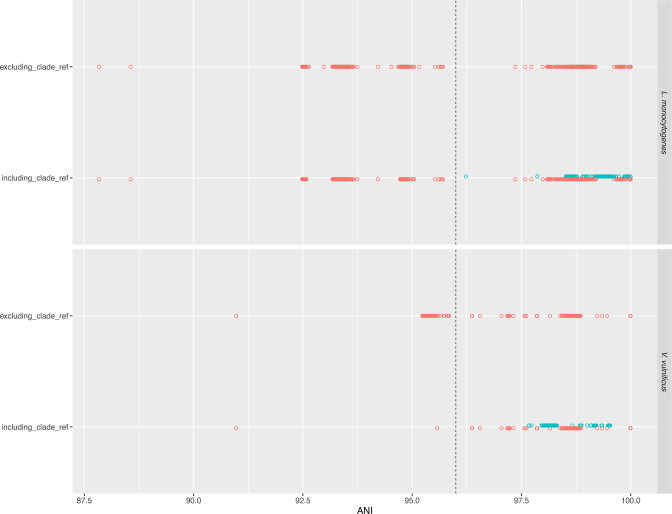
Adding additional representative assemblies for species with broad genomic diversity improves taxon identification. Intraspecies ANI of all assemblies from *Listeria monocytogenes* (* L. monocytogenes *) and *Vibrio vulnificus* (* V. vulnificus *) against only their corresponding type assemblies (‘excluding clade ref’) and after including additional representative assemblies (‘including clade ref’). Red circles indicate the best match ANI value of assemblies against only their type assemblies and blue circles indicate the new best match ANI value after including the additional representative assemblies. 8 % of *L. monocytogenes* and 99 % of *V. vulnificus* assemblies that previously didn’t match their type assemblies matched the newly added representative assemblies at above the expected ANI threshold.

Intraspecies genomic diversity can be addressed by lowering the ANI threshold that includes all assemblies from the species as defined or by considering additional representative assemblies in addition to the type assemblies or a combination of both. For species with subspecific clusters or groups, additional representative assemblies can be selected for each cluster or group whilst awaiting additional taxonomic work to label these formally as potentially novel species or subspecies. The Centres for Disease Control and Prevention (CDC), as part of the PulseNet project (https://www.cdc.gov/pulsenet/index.html), uses multiple representative assemblies for many species in order to ensure that this diversity is adequately addressed in computational comparisons. For example, there are at least two clusters for *Campylobacter lari* and CDC uses one representative (none of which are from the type strain) for each cluster (Fig. S3, personal communication). In this study, as a proof of concept, CDC representative assemblies from two species (* Vibrio vulnificus* and *Listeria monocytogenes *) were adopted as extra representatives in addition to type assemblies. Adding additional representatives improved the taxon identification for these two species. Fig. 3 shows the best match ANI values of all assemblies from these two species against (1) only their corresponding type assemblies and (2) after adding corresponding, representative assemblies. 99 % of the *V. vulnificus* and 8 % of the *L. monocytogenes* assemblies that previously did not match their corresponding type assemblies above the expected ANI threshold (96 %), matched their corresponding representative assemblies above the expected ANI threshold.

### ANI of some type assemblies do not support synonymizing or merging of two taxa

A species can only have one correct name (referred to as the ‘current name’ in NCBI Taxonomy) and one set of co-identical type strains. When two taxa that were described independently with separate type strain declarations are determined to be the same species, the earlier name will have precedence and the later one would be considered as a later heterotypic synonym of the initially described taxon. The type strains associated with the heterotypic synonym have no standing with regard to the correct species name. Conversely, on the basis of the results of new analysis, existing heterotypic synonyms have been reclassified back as independent species (e.g., *Bacillus axarquiensis* [14]). ANI can be used to assess the heterotypic synonymy relationship. If the species is correctly defined, assemblies from heterotypic synonyms (referred to as ‘syntype assemblies’) are expected to be highly similar (i.e., higher ANI and coverage) to the assemblies from type strains of their corresponding species.

**Fig. 4.**
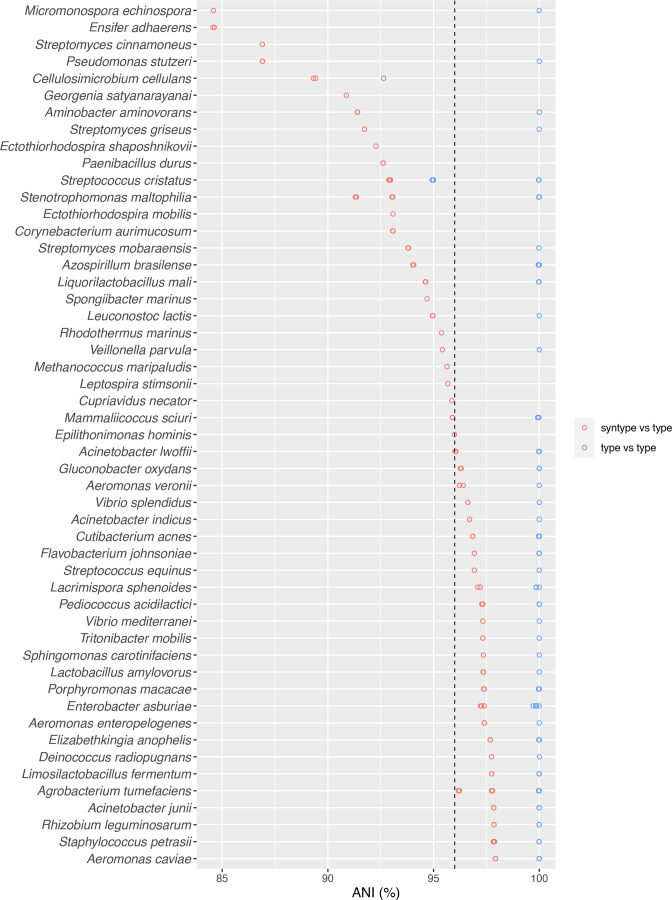
ANI-based verification of heterotypic synonymization. When two independently described taxa were determined to be the same species, the two taxa would be merged and the taxon that was described later would become the heterotypic synonym of the taxon that was described earlier. Lower ANI values of assemblies from heterotypic synonyms (referred to as ‘syntype assemblies’ or ‘syntype’) against the assemblies from type strains (referred to as ‘type assemblies’ or ‘type’) from the same species (red circles) indicate potentially problematic synonymizations. There were at least 27 cases where the ANI values of the assemblies from heterotypic synonyms were lower than the ANI threshold of the corresponding species. The dotted vertical line indicates the default ANI threshold of 96 %.

There are at least 257 taxa with assemblies from heterotypic synonyms and of these at least 220 have at least one assembly available from both type strains of their corresponding species and the heterotypic synonym. Fig. 4 shows the ANI values of intra-type assemblies (blue open circles) and type vs syntype assemblies (red open circles). There are at least 27 taxa for which the assemblies from heterotypic synonyms do not match the type assemblies of their corresponding species above the default ANI threshold of 96 (top 27 taxa in the Fig. 4). There are 26 taxa for which the assemblies from heterotypic synonyms do match above the ANI threshold, but the ANI value is lower than the ANI of the type vs type matches. Assuming there are no problems with the identification and sequencing of the assemblies considered, the synonymy of these taxa may need to be reconsidered.

### Species-specific ANI threshold

A default ANI threshold of 96 % has been considered to be enough to distinguish between assemblies from two different species [5, 15]. However, there have been cases where a threshold higher or lower than the 96 % threshold was required to define species boundaries. Using the automated process described in the methods, it was possible to propose custom ANI thresholds for 67 taxa (Fig. S4). For example, multiple assemblies from *Spiroplasma melliferum* match the type assembly of *Spiroplasma citri* above the default 96 % ANI threshold and using a higher ANI threshold of 98.8 % for *S. citri* separates these two species of the genus *Spiroplasma* (Fig. S4). Similarly, several assemblies from *Mycoplasma mycoides* match the type assemblies from their own species below the default 96 % ANI threshold. By lowering the ANI threshold for *M. mycoides* to 94.4%, all assemblies from the species match the type assemblies (Fig. S4). A full list of species and their custom ANI thresholds is available as an FTP file (https://ftp.ncbi.nlm.nih.gov/genomes/ASSEMBLY_REPORTS/ prokaryote_ANI_species_specific_threshold.txt).

### ANI-based validation of assemblies submitted to GenBank using type assemblies

Type assemblies were used as references for validating or reclassifying the taxonomy of all assemblies submitted to GenBank. The taxonomic check status for more than 1.1 million assemblies is summarized in the FTP file (https://ftp.ncbi.nlm.nih.gov/genomes/ASSEMBLY_REPORTS/ANI_report_prokaryotes.txt). In the last 5 years, the taxonomy of over 7000 newly sequenced genomes has been reclassified before acceptance to GenBank as has been the taxonomy of 1800 genomes that are already in GenBank. The taxonomy of 137 000 existing GenBank genomes cannot be validated as there is not sufficient data.

### Conclusions

The large-scale availability of prokaryotic genomes has already influenced prokaryotic taxonomy in a major way, forcing a shift away from a classification largely based on 16S sequences [16, 17] and opening up the possibility of adapting practices [18] in order to sample and reference microbial diversity comprehensively [19]. A stable, genome-based classification requires well validated references. In this paper, we have highlighted several processes introduced, over time, to annotate, validate and correct type genome assemblies, relying on the published literature, authoritative online resources and expert input as far as possible. Several additional processes at NCBI are available to improve data during the submission process including recent improvements in the Prokaryotic Genome Annotation Pipeline (PGAP [20]) that allow for pre-submission verifications. Nevertheless, improving and accurately filtering out imprecise and inaccurate content remains a daunting task and users should always treat available data with healthy scepticism and perform appropriate verifications where possible [21, 22]. The use of type genomes remains central to any of these actions and continued vigilance and adjustments are needed to ensure major errors are kept to a minimum. In some cases, the limitations of type genomes are evident and additional adjustments are needed to address underlying genetic variation. At the same time, prokaryotic taxonomy is undergoing major changes, specifically the treatment of uncultured, environmentally sampled genomes, which do not have well fleshed out nomenclature, is posing challenges for data re-use [23]. Proposals to deal with uncultivated taxa range from utilizing a separate code of nomenclature, the SeqCode [24] to extending the use of *Candidatus* names [25], possibly by utilizing algorithms creating neutral latinized labels that follow current grammatical rules [26]. Some of these proposals have already been debated and not incorporated into the ICNP [27]. Nevertheless, curators and archivists at the public sequence repositories will have to follow any discussion closely and consider adopting and improving ANI and related processes if any broad consensus emerges.

There is a range of resources available online supporting nomenclature and taxonomy of prokaryotes (listed in [16]). As a central depository of public genome sequences that also serves multiple other resources and uses, we believe the most important part of our actions in NCBI Taxonomy and Assembly resources should be devoted to making the taxonomic annotation associated with type assemblies as accurate as possible, while continuing to extend and improve on the processes described here. With the existing curated type assemblies and ANI-based processes we were able to reclassify the taxonomy of over 8800 non-type assemblies during and post submission to GenBank. However, there are still many species without any assemblies from type strains and we have highlighted the high priority candidates for sequencing. We welcome input from the research community to improve our ANI-based curation of public type and non-type assemblies.

## Supplementary Data

Supplementary material 1
