# Supplementary material for: Collection and curation of prokaryotic genome assemblies from type strains at NCBI
Source: Int J Syst Evol Microbiol. 2023 Jan 19;73(1):005707. doi: 10.1099/ijsem.0.005707 (PMC10228379; doi:10.1099/ijsem.0.005707)
Supplement: Supplementary material 1 [file ijsem-73-5707-s001.pdf]

Supplementary information

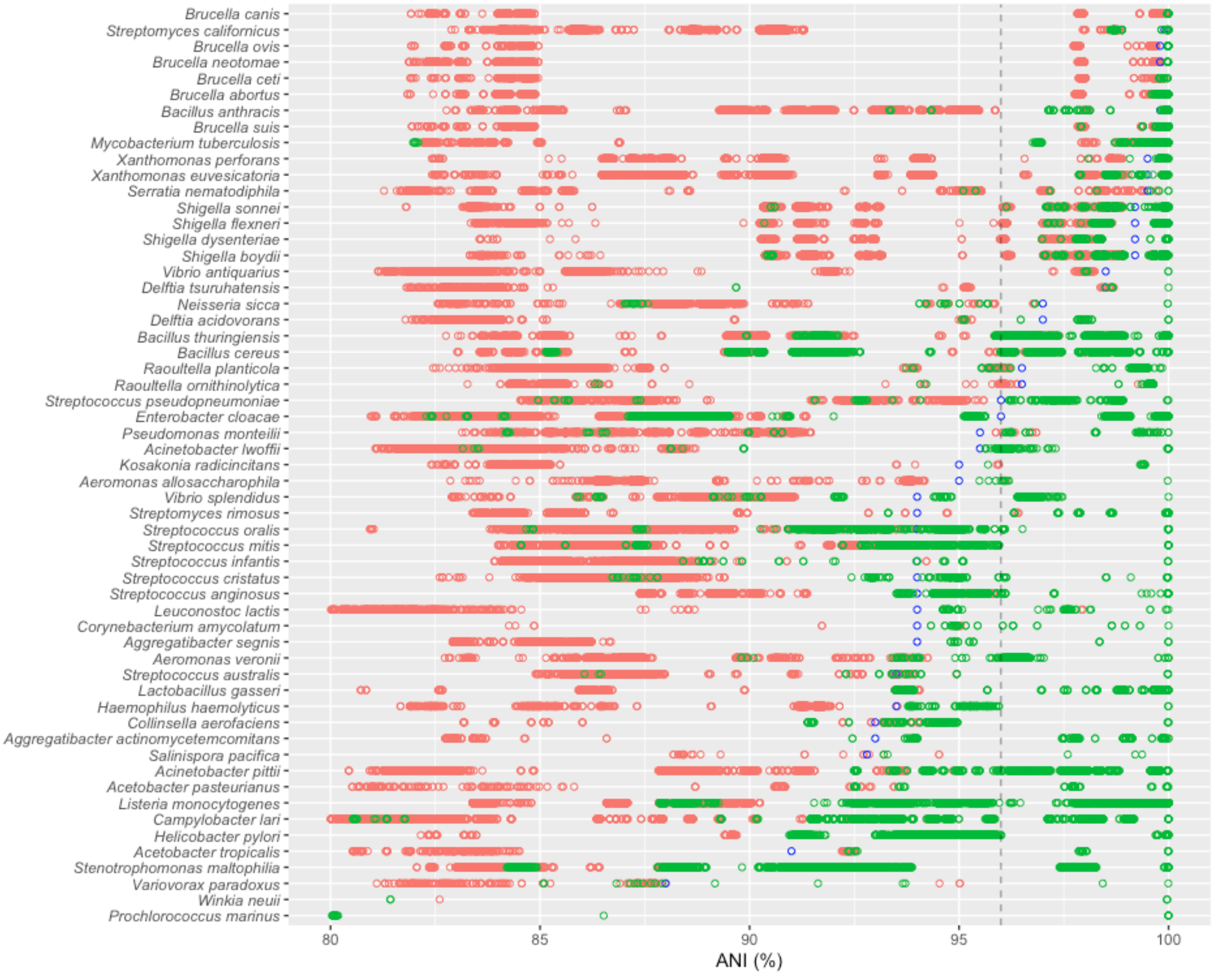

Figure S1. Default (gray vertical line) and manually selected ANI threshold (blue circle) for 79 species. Green and red circles indicate intra- and inter-species ANI values against the type assemblies from the species of interest (y-axis).

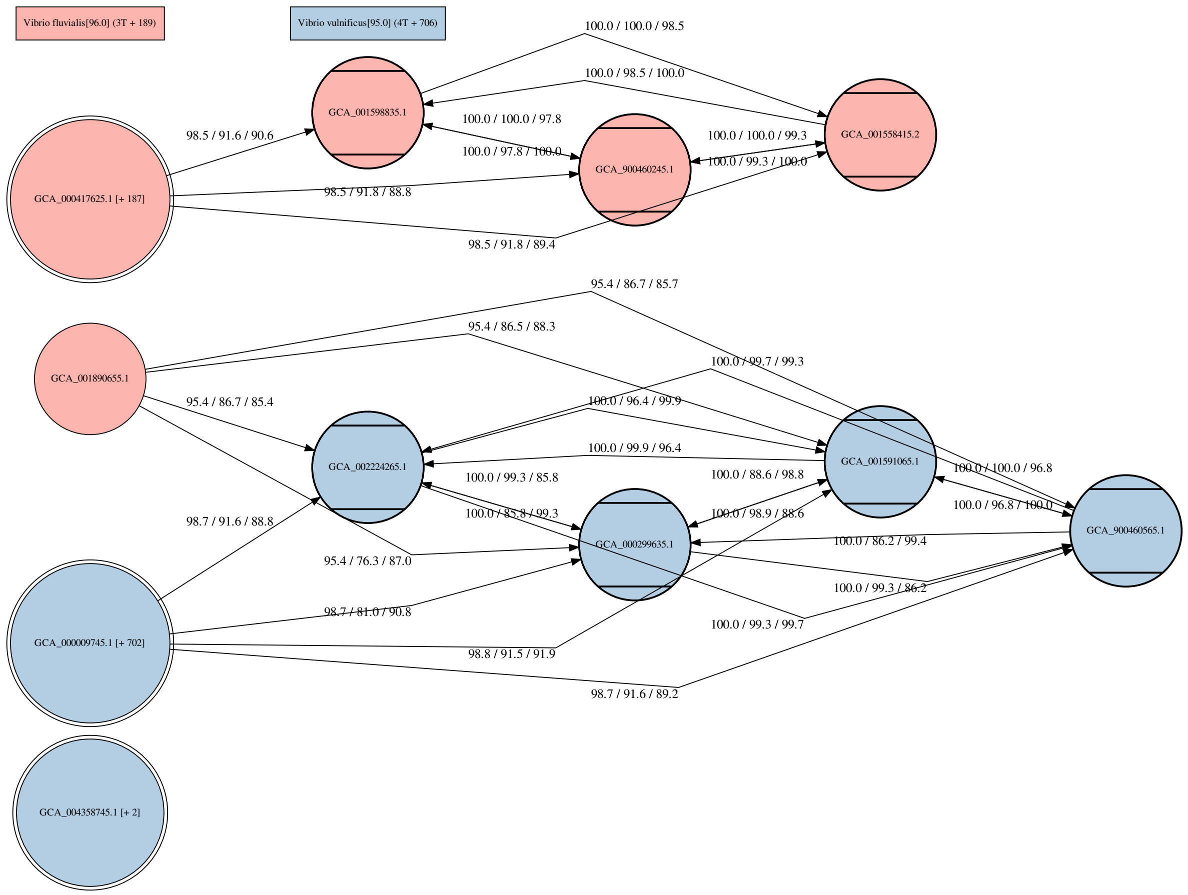

Figure S2. A problematic type assembly (accession: GCA_001890655.1) from *Vibrio fluvialis*. The figure shows the ANI, query and subject coverage values (in the arrows) for the assemblies from *Vibrio fluvialis* (red color circles) and *Vibrio vulnificus* (blue color circles) against the type assemblies (circles with parallel lines inside the circle). Non-type assemblies are shown as circles without parallel lines. Non-type assemblies from the same species that match the same type assemblies or do not match any type assemblies were grouped and shown in concentric circles (for example, the concentric circles with the label GCA_004358745.1 [+ 2] indicate that the assembly GCA_004358745.1 and two other assemblies from *V. vulnificus* do not match any type assemblies). The *V. fluvialis* type assembly of interest, GCA_001890655.1 did not match the other three type assemblies from its own species but matched all the four type assemblies from a different species, *V. vulnificus*. This type assembly is most likely misidentified.

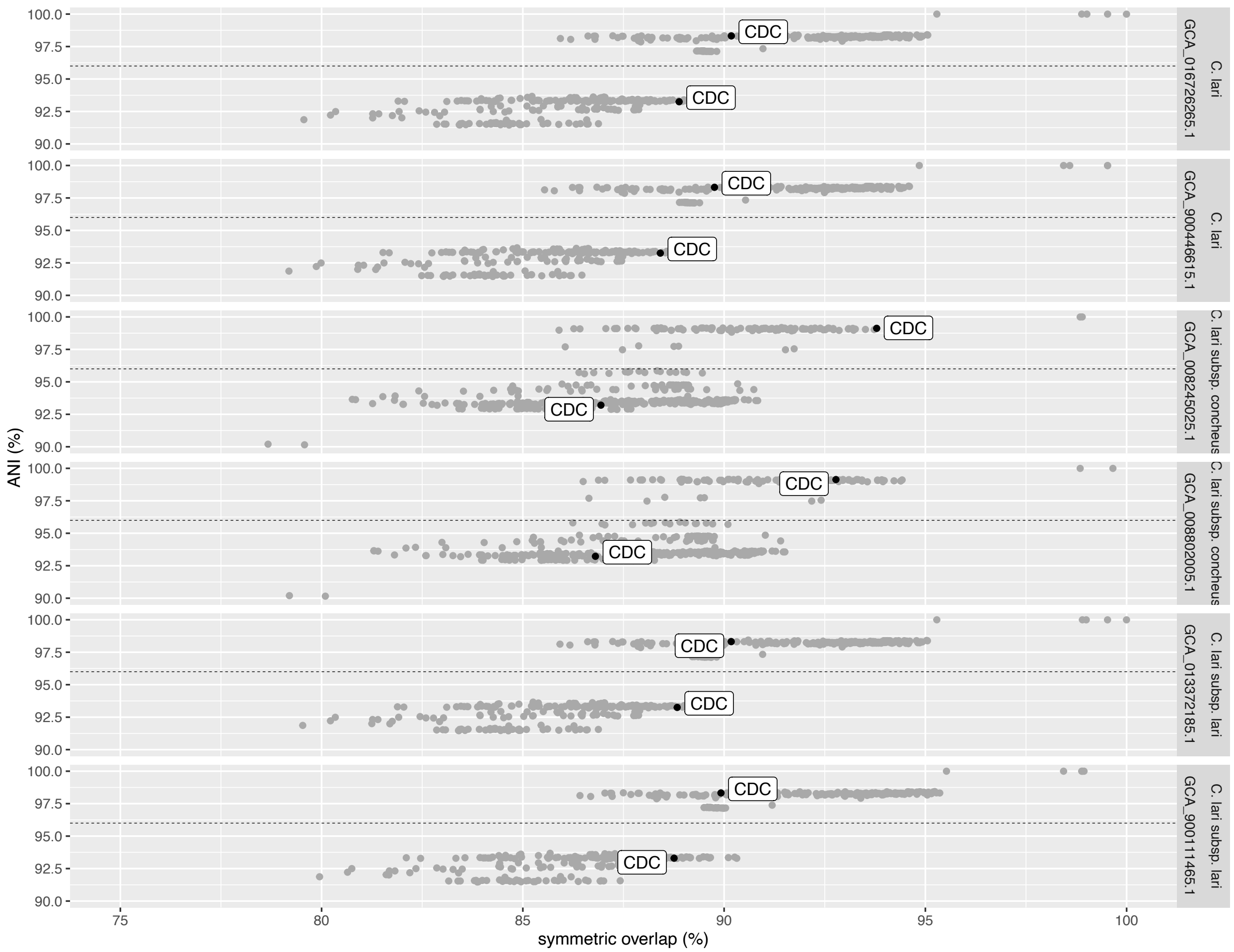

Figure S3. **Addressing intraspecies genomic diversity by using multiple representatives in computational comparisons.**

Intraspecies ANI vs symmetric overlap (matched region length over the total length among a pair of assemblies) of assemblies against the type assemblies from *Campylobacter lari*. Horizontal dotted line indicates the default ANI threshold, 96%. The Centers for Disease Control and Prevention (CDC) as part of the PulseNet project (https://www.cdc.gov/pulsenet/index.html), uses multiple representative assemblies (highlighted in the figure) for *Campylobacter lari* in order to ensure that this diversity is adequately addressed in computational comparisons.

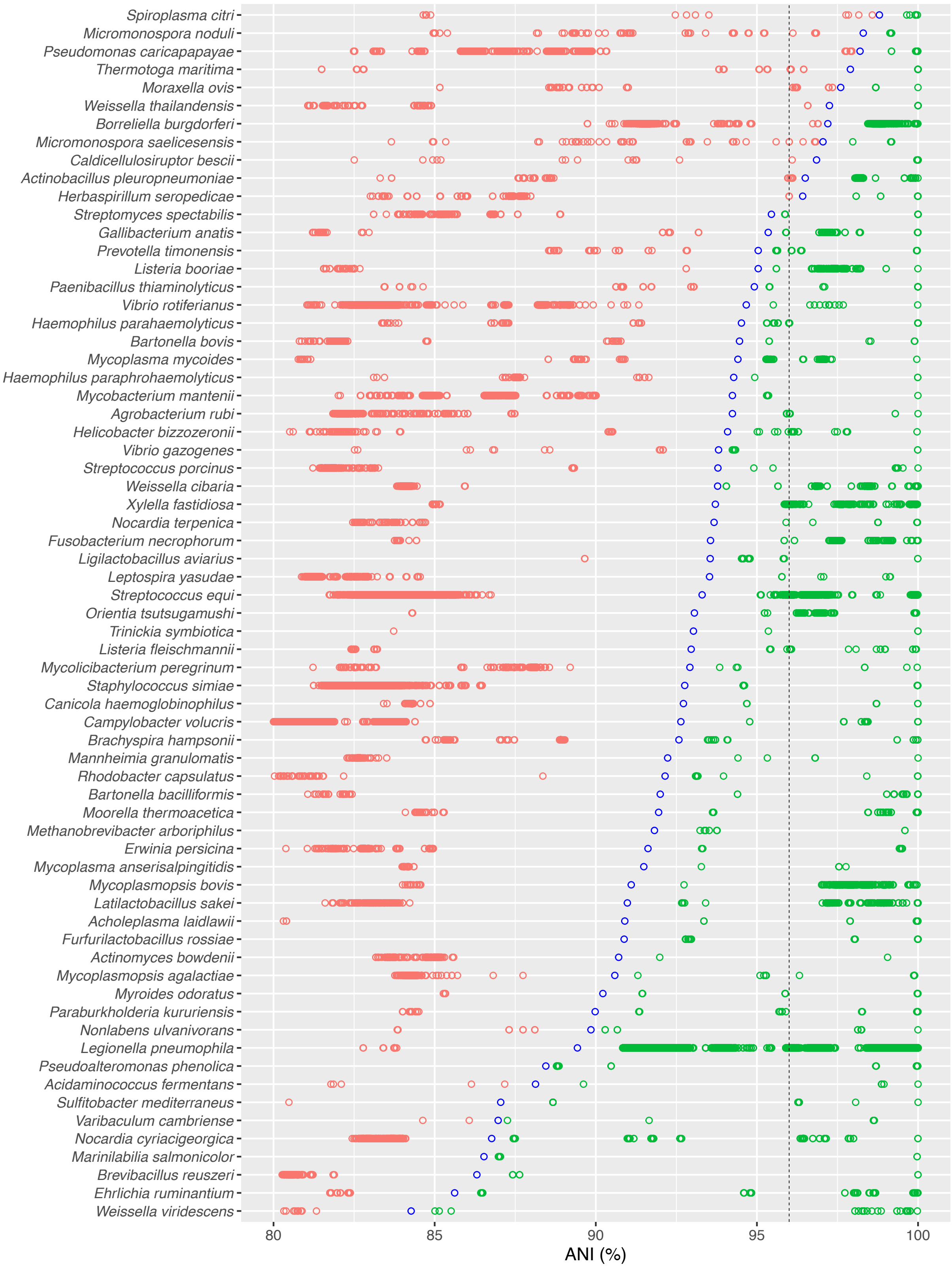
Spiroplasma citri
Micromonospora noduli
Pseudomonas caricapapayae
Thermotoga maritima
Moraxella ovis
Weissella thailandensis
Borreliella burgdorferi
Micromonospora saelicesensis
Caldicellulosiruptor bescii
Actinobacillus pleuropneumoniae
Herbaspirillum seropedicae
Streptomyces spectabilis
Gallibacterium anatis
Prevotella timonensis
Listeria booriae
Paenibacillus thiaminolyticus
Vibrio rotiferianus
Haemophilus parahaemolyticus
Bartonella bovis
Mycoplasma mycoides
Haemophilus paraphrohaemolyticus
Mycobacterium mantenii
Agrobacterium rubi
Helicobacter bizzozeronii
Vibrio gazogenes
Streptococcus porcinus
Weissella cibaria
Xylella fastidiosa
Nocardia terpenica
Fusobacterium necrophorum
Ligilactobacillus aviarius
Leptospira yasudae
Streptococcus equi
Orientia tsutsugamushi
Trinickia symbiotica
Listeria fleischmannii
Mycolicibacterium peregrinum
Staphylococcus simiae
Canicola haemoglobinophilus
Campylobacter volucris
Brachyspira hampsonii
Mannheimia granulomatis
Rhodobacter capsulatus
Bartonella bacilliformis
Moorella thermoacetica
Methanobrevibacter arboriphilus
Erwinia persicina
Mycoplasma anserisalpingitidis
Mycoplasmopsis bovis
Latilactobacillus sakei
Acholeplasma laidlawii
Furfurilactobacillus rossiae
Actinomyces bowdenii
Mycoplasmopsis agalactiae
Myroides odoratus
Paraburkholderia kururiensis
Nonlabens ulvanivorans
Legionella pneumophila
Pseudoalteromonas phenolica
Acidaminococcus fermentans
Sulfitobacter mediterraneus
Varibaculum cambriense
Nocardia cyriacigeorgica
Marinilabilia salmonicolor
Brevibacillus reuszeri
Ehrlichia ruminantium
Weissella viridescens
80
85
90
95
100
ANI (%)

Figure S4. Default (black vertical line) and automatically determined new ANI threshold (blue circle) for 67 species. Green and red circles indicate intra- and inter-species ANI values against the type assemblies from the species of interest (y-axis).

1. Assembly anomalies and other reasons an assembly from type may be excluded

contaminated - sequences from another organism, cloning vectors, linkers, adapters or primers are present in the assembly.

derived from single cell - the source material for the assembly was amplified from a single cell leading to concern about the genome sequence accuracy.

genome length too large - total non-gapped sequence length of the assembly is more than 1.5 times that of the average for the genomes in the Assembly resource from the same species, more than 15 Mbp, or is otherwise suspiciously long.

genome length too small - total non-gapped sequence length of the assembly is less than half that of the average for the genomes in the Assembly resource from the same species, less than 300 Kbp, or is otherwise suspiciously short.

genus undefined - the lineage does not include a genus, hence, the precise taxonomic placement is uncertain. An exception is made for symbionts.

low quality sequence - long stretches of the sequence have a high proportion of ambiguous bases, are low complexity, or have some other indication that the sequence quality is low.

misassembled - alignment to related genome assemblies or other evidence indicates the assembly is likely to have errors.

missing strain identifier - prokaryote assembly lacking both strain and isolate identifiers in the appropriate field. Exceptions are made for symbionts and phytoplasmas.

mixed culture - sequences come from two or more organisms that were not cultured separately.

partial - the assembly has only partial genome representation.

unverified source organism - the origin of the assembly is misidentified.

2. Assembly anomalies and other reasons an assembly from type may still be considered

derived from metagenome - the genomic sequence was assembled from metagenomic sequencing rather than a pure culture leading to concerns about the accuracy of organism assignment and possible cross-contamination.

derived from environmental source - the source material for the assembly is from an environmental source rather than a pure culture leading to concern about the accuracy of organism assignment and possible cross-contamination.

fragmented assembly - a prokaryotic assembly with contig L50 above 500, contig N50 below 5000, or more than 2,000 contigs.

3. *Assembly level* - the highest level of assembly for any object in the assembly:

- *Complete genome* - all chromosomes are gapless and have no runs of 10 or more ambiguous bases (Ns), there are no unplaced or unlocalized scaffolds, and all the expected chromosomes are present (i.e. the assembly is not noted as having partial genome representation). Plasmids and organelles may or may not be included in the assembly but if present then the sequences are gapless.
- *Chromosome* - there is sequence for one or more chromosomes. This could be a completely sequenced chromosome without gaps or a chromosome containing scaffolds or contigs with gaps between them. There may also be unplaced or unlocalized scaffolds.
- *Scaffold* - some sequence contigs have been connected across gaps to create scaffolds, but the scaffolds are all unplaced or unlocalized
- *Contig* - nothing is assembled beyond the level of sequence contigs
